# Letter to the Editor: Cemented versus uncemented hemiarthroplasty for femoral neck fractures in patients with neuromuscular diseases: a minimum of 2 years’ follow-up study

**DOI:** 10.1186/s13018-021-02670-5

**Published:** 2021-08-20

**Authors:** Wang Xing, Huang Qiang, Pei Fuxing

**Affiliations:** Department of Orthopaedic Surgery, West China Hospital, Sichuan University, No. 37, Guoxue Road, Wuhou district, Chengdu, 610041 Sichuan People’s Republic of China

Dear Editor,

Recently, I read with great interest the study entitled “Cemented versus uncemented hemiarthroplasty for femoral neck fractures in patients with neuromuscular diseases: a minimum of 2 years’ follow-up study” by Wang et al. [1] The following concerns need to be explained cautiously.

First, authors did not mention the time points and frequency of pain evaluation. The same problem existed with Harris hip score (HHS). Authors found the intraoperative blood loss were less in the uncemented group. However, they did not mention how was intraoperative blood loss medical calculated? Furthermore, authors reported that although the between-group difference was not statistically significant, they found that the uncemented group was associated with higher risk of postoperative periprosthetic fracture. In addition to bone density, periprosthetic fractures are generally related to either inexperienced surgeons or inadequate surgical technique. However, they did not mention were all surgeries performed by same surgeon with standard procedure? If there is a difference between the two groups, it will have a certain impact on the effect of surgery and complications.

Second, the authors concluded that intraoperative death almost exclusively occurred during cemented procedures, which may be caused by the Bone cement implantation syndrome (BCIS), but no intraoperative death was found in this study, which showed that the use of cement has no detrimental effect on the short- and mid-term mortality in patients with neuromuscular diseases. However, BCIS is rare, and so historically controlled comparative studies are difficult, because the infrequency of the event makes it hard to compare risk factors relevant to the condition. However, large national databases have given us populations to perform reliable statistical analyses [2]. In a study of more than 11,000 patients with hip fracture, they found use of cement to be independently associated with an increased risk of death on the day of surgery and the following day. When ASA increased from 1 or 2 to 3 or 4, the number needed to harm (to cause 1 death from cementation) rose from 1 of 811 patients to 1 of 33 operated patients [2]. About 60% of these early fatalities were directly attributed to bone cement [3]. Thus, the key to avoiding cement-related deaths during or soon after hemiarthroplasty surgery for hip fracture is for the surgeon to choose an uncemented implant.

## Data Availability

Not applicable.

## Abbreviations

VAS: Visual analog scale
HHS: Harris hip score
BCIS: Bone cement implantation syndrome
ASA: American Society of Anesthesiologists
